# Factors associated with difficulty in hospital acceptance during the COVID-19 pandemic period in Osaka Prefecture, Japan: a population-based study

**DOI:** 10.3389/fpubh.2024.1391519

**Published:** 2024-05-30

**Authors:** Yusuke Katayama, Kenta Tanaka, Shunichiro Nakao, Jotaro Tachino, Tomoya Hirose, Hisaya Dohmi, Tetsuhisa Kitamura, Jun Oda, Tetsuya Matsuoka

**Affiliations:** ^1^The Working Group to Analyze the Emergency Medical Care System in Osaka Prefecture, Osaka, Japan; ^2^Department of Traumatology and Acute Critical Medicine, Osaka University Graduate School of Medicine, Suita, Japan; ^3^Department of Social and Environmental Medicine, Division of Environmental Medicine and Population Sciences, Osaka University Graduate School of Medicine, Suita, Japan; ^4^Osaka Prefectural Government, Osaka, Japan; ^5^Rinku General Medical Center, Izumisano, Japan

**Keywords:** emergency medical service, COVID-19, pandemic, prognosis, public health, epidemiology

## Abstract

**Background:**

In many countries, emergency medical systems were responsible for initial treatment of patients with COVID-19. Generally, acceptance by medical institutions may not be sufficient, and it may take much time to determine the medical institution to which to transport the patient. This problem is termed “difficulty in hospital acceptance (DIH),” and it is used as a key performance indicator in the assessment of the EMS in Japan. The purpose of this study was to reveal the factors associated with the DIH during the COVID-19 pandemic using dataset in the ORION (Osaka emergency information Research Intelligent Operation Network system).

**Methods:**

This was a retrospective descriptive study with a 3-year study period from January 1, 2019 to December 31, 2021. We included patients who were recorded in the ORION system during the study period. The primary endpoint was defined as DIH. Multivariable logistic regression model was used to assess factors associated with DIH during the COVID-19 pandemic and calculated their adjusted odds ratio (AOR) and associated 95% confidence interval (CI).

**Results:**

1,078,850 patients included in this study. Of them, 41,140 patients (3.8%) experienced DIH and 1,037,710 patients (96.2%) did not experience DIH. The median age was 71 years (IQR: 45–82), and 543,760 patients (50.4%) were male. In this study, SpO_2_, body temperature, and epidemic period of COVID-19 were associated with difficulty in hospital acceptance. The highest AOR of SpO2 was 80% or less (AOR: 1.636, [95% CI: 1.532–1.748]), followed by 81–85% (AOR: 1.584, [95% CI: 1.459–1.721]). The highest AOR of body temperature was 38.0–38.9°C (AOR: 1.969 [95% CI: 1.897–2.043]), followed by 39°C or higher (AOR: 1.912 [95% CI: 1.829–1.998]). The highest AOR of epidemic period of COVID-19 was the 4th wave (AOR: 2.134, [95% CI: 2.065–2.205]), followed by the 3rd wave (AOR: 1.842, [95% CI: 1.785–1.901]).

**Conclusion:**

In this study, we revealed factors associated with the DIH during the COVID-19 pandemic. As various factors are involved in the spread of an unknown infectious disease, it is necessary not only to plan in advance but also to take appropriate measures according to the situation in order to smoothly accept emergency patients.

## Introduction

Novel coronavirus (COVID-19) was first identified in Wuhan, China, in December 2019, and has since spread worldwide, including to Japan (1–7). COVID-19 is an infectious virus that causes severe respiratory failure. The patient with COVID-19 requires respiratory assistance and extracorporeal membrane oxygenation (ECMO), and emergency medical systems were responsible for initial treatment of patients with COVID-19 in many countries. Initially, because the infection route of COVID-19 was unknown, many medical workers such as physicians and nurses were required to take strict infection precautions, and these strict infection precautions affected the treatment of non-COVID-19 patients. However, as the pathogenesis of COVID-19 was revealed and the development of vaccines and vaccination against COVID-19 progressed, infection precautions against COVID-19 were phased out worldwide. In Japan, the Infectious Disease Control Law was revised in May 2023, with the infection precautions against COVID-19 lifted, and the usual medical care system has been recovered (8).

In Japan, the Emergency Medical System (EMS) is a public service, and patients can call for an ambulance free of charge. After the patient calls, the patient is evaluated by EMS personnel at the scene, and the appropriate medical institution is selected to which to transport the patient based on that evaluation. The EMS personnel at the scene negotiate with doctors and nurses in the emergency medical institutions for permission to transport the patient. However, depending on the patient’s condition and the time of day when the patient called for an ambulance, acceptance rates differ by institutions, and acceptance by the nearest medical institution cannot be assumed; there may be delays in determining the institution to which to transport the patient. This problem is termed “difficulty in hospital acceptance (DIH),” and it is used as a key performance indicator in the assessment of the EMS in Japan. We have previously identified factors related to DIH (9). However, it remains unclear whether the epidemic period of COVID-19 infection or patient status affected the DIH during the COVID-19 pandemic period. Review of this pandemic and determining the impact of the COVID-19 pandemic on the EMS are critical when considering policies against infectious disease pandemics in the future.

Osaka Prefecture, the largest metropolitan area in western Japan, has a population of 8.8 million people and generates approximately a half million calls for ambulances each year (10). Since the first patient with COVID-19 was identified in Osaka Prefecture on January 23, 2020, the cumulative number of COVID-19 patients in Osaka Prefecture as of December 31, 2021 was 203,790 (11). In Osaka Prefecture, emergency patients transported by ambulance have been registered in the ORION system since 2015 (12, 13). The purpose of this study was to reveal the factors associated with the DIH during the COVID-19 pandemic using ORION data.

## Materials and methods

### Study design and settings

This was a retrospective descriptive study with a 3-year study period from January 1, 2019 to December 31, 2021. We included patients who were recorded in the ORION system during the study period. Therefore, exclusion criteria for this study were cases with missing data and inter-hospital transfer cases.

In 2020, 8,837,685 people lived in the 1905 km^2^ area of Osaka Prefecture. Of that population, 4,235,956 people (47.9%) were male and 2,441,984 people (25.4%) were considered older adult, aged 65 years old or more (10). Because the ORION data is anonymized without specific personal data, such as patient name, date of birth, and address, the requirement of obtaining patients’ informed consent was waived. This study was approved by the Ethics Committee of Osaka University Graduate School of Medicine, Suita, Japan (approval number: 15003). This manuscript was written based on the STROBE statement to assess the reporting of cohort and cross-sectional studies (14).

### Ems system and hospitals in Osaka Prefecture

The EMS system is basically the same as that used in other areas of Japan. In Osaka Prefecture, EMS systems such as ambulance dispatch systems are operated by each local government, and ambulances are dispatched by calling 1–1–9. In 2021, the EMS system was operated by 26 fire departments (298 ambulances) and 26 fire control stations. In 2018, there were 517 medical institutions (105,994 beds) in Osaka Prefecture (15), of which 288 are emergency medical hospitals including 16 critical care centers that are designated to accept patients with life-threatening emergency diseases such as severe trauma and sepsis. Since the introduction of the ORION system, EMS personnel at the scene select the appropriate hospital for emergency patients rather than a dispatcher.

### The ORION system

Information on the system configuration of ORION was previously described in detail (12, 13). The EMS personnel at the scene operate the ORION smartphone app for each emergency patient. All of the data input into this cellphone app, such as vital signs and the time of the call to the hospital for acceptance, are also recorded. The cellphone app data are accumulated in the ORION cloud server, and in cooperation with the dispatched EMS personnel, data managers at each fire department directly input or upload the ambulance record of each emergency patient so that it can be connected with the app data. Furthermore, the operators of each hospital also directly input or upload the patient’s data, such as diagnoses and outcomes, after hospital acceptance. The results of the aggregated data in the ORION system are fed back to every fire department and emergency hospital. The Department of Public Health of Osaka Prefecture can also analyze the effects of health policy on the emergency medical system using these collected data. The ORION system has been in place in all fire departments and emergency hospitals in Osaka Prefecture since January 2016.

### The COVID-19 pandemic in Osaka Prefecture

**Figure 1:**
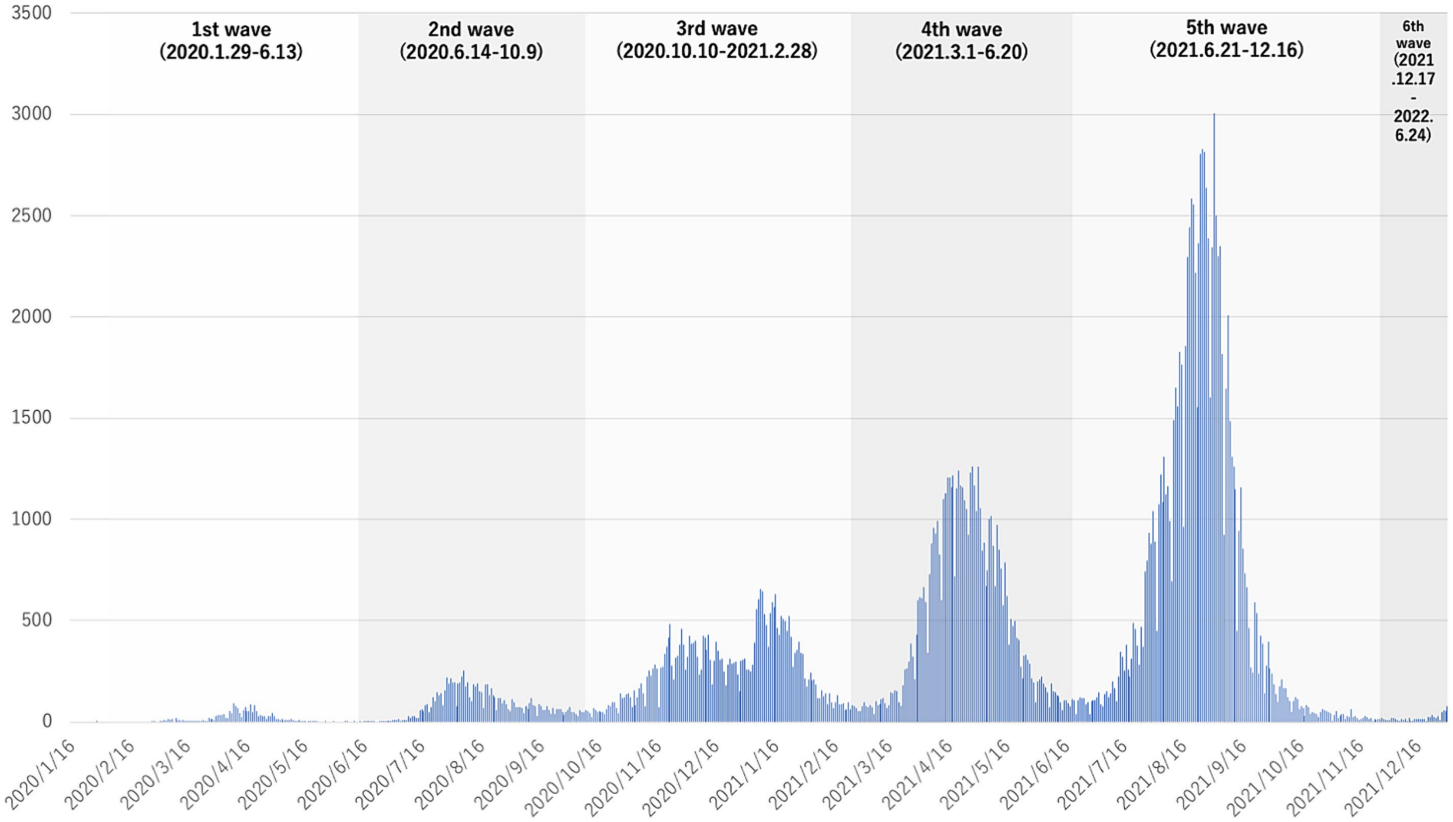
The number of patients with COVID-19 per week during the study period.

We have previously revealed the characteristics and outcome of patients with COVID-19 in Osaka Prefecture (16). In Japan, based on the Infectious Diseases Control Law, patients diagnosed as having COVID-19 using a polymerase chain reaction (PCR) test or antigen test at medical institutions were reported to the public health department and the number of patients was counted and published until May 2023 (8). In Osaka Prefecture, as in other countries, the number of patients with COVID-19 increased as the genetic form of COVID-19 changed (11). The public health department took the lead in arranging medical institutions for patients diagnosed as having COVID-19 who required inpatient care. As the number of COVID-19 patients increased and it became difficult to provide inpatient care, doctors and nurses were assigned to loading facilities such as hotels, and these facilities were used as temporary medical facilities to accommodate COVID-19 patients. In Osaka Prefecture, the epidemic period of COVID-19 infection was defined as the first wave (1/29/2020–6/13/2020), second wave (6/14/2020–10/9/2020), third wave (10/10/2020–2/28/2021), fourth wave (3/1/2021–6/20/2021), fifth wave (6/21/2021–12/16/2021), and sixth wave (12/17/2021–6/24/2022) based on the number of patients newly infected with COVID-19 (Figure 1) (17). Because we used an annual data set in this study, we only included 2 weeks for the six wave (12/17/2021–12/31/2021).

### Data collection and quality control

The ORION system checks for errors in the inputted in-hospital data, and the staff of each emergency hospital can correct them if necessary. Through these tasks, cellphone app data, ambulance records, and the in-hospital data such as diagnosis and prognosis can be comprehensively registered for each patient transported by an ambulance. The registered data is cleaned by the Working Group to analyze the emergency medical care system in Osaka Prefecture (12). Among the collected and cleaned data, we excluded inconsistent data that did not contain all of the cellphone app data, ambulance records, and in-hospital data such as diagnosis and prognosis. In addition, we also excluded patients whose sex as registered by the fire department did not match that registered by the hospital or whose sex was missing. We also excluded patients whose age input by the fire department and that by the hospital differed by 3 years or more. When this difference was present, we defined the age input by the hospital as the patient’s true age.

### Endpoints

The primary endpoint was defined as DIH. In this study, DIH was defined as a case in which the patient stayed at the scene for more than 30 min and required more than 4 attempts to determine which medical institution to transport to based on the definition by the Fire and Disaster Management Agency (18). The secondary endpoint was mortality of patients with DIH for each COVID-19 epidemic period. Mortality was calculated as the percentage of patients who died within 21 days of ambulance transport among the patients hospitalized after ambulance transport.

### Statistical analysis

In this study, we used a multivariable logistic regression model to assess factors associated with DIH during the COVID-19 pandemic and calculated their adjusted odds ratio (AOR) and associated 95% confidence interval (CI). A multivariable logistic regression model was conducted using forced entry methods. Based on a previous study (9), potential covariates were age group, gender, saturation of oxygen (SpO_2_), disturbance of consciousness, body temperature (BT), time of day, day of the week, place of occurrence, reason for ambulance call, and the epidemic period of COVID-19. Because we hypothesized that the suggested COVID-19 infection would affect hospital acceptance, we entered body temperature and SpO_2_ into the regression model as explanatory variables. Age groups were classified into children (0–14 years), adults (15–64 years) and older adult (over 65 years). SpO_2_ was classified in 5% increments, and those below 80% were integrated. BT was classified in 1°C increments, and those above 39°C and below 34°C were merged. Disturbance of consciousness was classified by Glasgow Coma Scale (GCS) and classified as coma (GCS: 3–8) and non-coma (GCS: 9–15). Time periods were classified as daytime (8:00–17:59) and nighttime (0,00–7:59, 18:00–23:59). Reason for ambulance call was classified into “Fire accident,” “Natural disaster,” “Water accident,” “Traffic accident involving car, ship, or aircraft,” “Injury, poisoning, and disease due to industrial accident,”” Disease and injury due to sports,”” Other injury,”” Trauma due to assault,” “Self-induced injury,” and “Acute disease.” The COVID-19 epidemic period was classified based on the definition by Osaka Prefecture. In addition, as a subgroup analysis, ORs and 95% CIs for potential covariates were calculated using the multivariate logistic regression model separately for each COVID-19 epidemic period. Data are presented as medians and interquartile ranges (IQR) for continuous variables and as percentages for categorical variables. Statistically significant differences were defined as those with *p* < 0.05, and SPSS Statistics ver. 27.0 J (IBM) was used as the statistical software.

## Results

**Figure 2:**
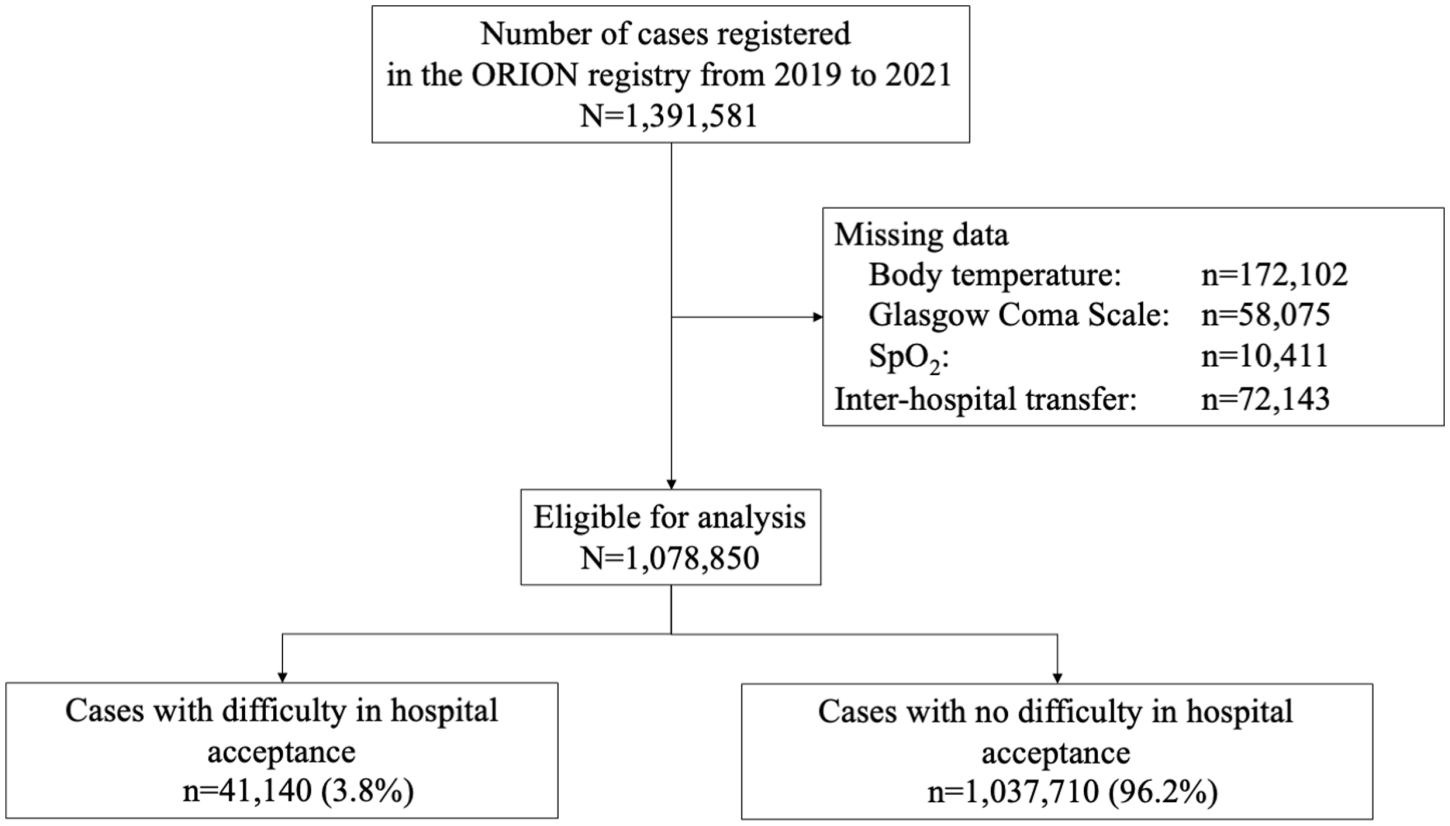
The patient flow in this study.

Figure 2 shows the patient flow in this study. During the study period, 1,391,581 patients were registered in the ORION system. Patients with missing data (BT: *n* = 172,102, GCS: *n* = 58,075, SpO_2_: *n* = 10,411) and inter-hospital transfer cases (*n* = 72,143) were excluded, resulting in 1,078,850 patients being included in this study. Of them, 41,140 patients (3.8%) experienced DIH and 1,037,710 patients (96.2%) did not experience DIH.

Table 1 shows the characteristics of the study patients. The median age was 71 years (IQR: 45–82), 68,765 (6.4%) were children, 625,431 (58.0%) were adults, 384,654 (35.7%) were older adults, 543,760 (50.4%) were male, and 535,090 (49.6%) were female. The SpO_2_ was 86–90% in 28,627 patients (2.7%), 81–85% in 10,021 patients (0.9%), and below 80% in 16,134 patients (1.5%), and 25,742 patients (2.4%) were in a coma. Among the patients with fever, the BT was 37.0–37.9°C in 195,335 patients (18.1%), 38.0–38.9°C in 63,574 patients (5.9%), and ≥ 39.0°C in 49,817 patients (4.6%). The most common calling location was home (696,057, 64.5%), followed by a public space (191,160, 17.7%). The most common reason for the ambulance call was “acute disease” (786,416, 72.9%), followed by “other injury” (180,226, 16.7%). Regarding the epidemic of COVID-19, the number of patients in the pre-pandemic period was 425,768 (39.5%), with 121,247 (11.2%) in the 1st wave, 117,787 (10.9%) in the 2nd wave, 127,181 (11.8%) in the 3rd wave, 97,417 (9.0%) in the 4th wave, 174,174 (16.1%) in the 5th wave, and 15,276 (1.4%) in the 6th wave.

**Table 1:** Patient characteristics.

	Total		Difficulty in hospital acceptance		No difficulty in hospital acceptance	
	(*n* = 1,078,850)		(*n* = 41,140)		(*n* = 1,037,710)	
Age, years, median (IQR)	71	(45–82)	69	(45–82)	71	(45–82)
Age groups, *n* (%)						
Children (0–14 years old)	68,765	(6.4)	956	(2.3)	67,809	(6.5)
Adult (≥18 years, <65 years)	6,25,431	(58.0)	22,825	(55.5)	6,02,606	(58.1)
Older adult (≥65 years)	3,84,654	(35.7)	17,359	(42.2)	3,67,295	(35.4)
Sex, *n* (%)						
Male	5,43,760	(50.4)	21,879	(53.2)	5,21,881	(50.3)
Female	5,35,090	(49.6)	19,261	(46.8)	5,15,829	(49.7)
Saturation of oxygen (SpO_2_)						
96–100%	8,89,012	(82.4)	31,740	(77.2)	8,57,272	(82.6)
91–95%	1,35,056	(12.5)	6,003	(14.6)	1,29,053	(12.4)
86–90%	28,627	(2.7)	1,704	(4.1)	26,923	(2.6)
81–85%	10,021	(0.9)	643	(1.6)	9,378	(0.9)
≤80%	16,134	(1.5)	1,050	(2.6)	15,084	(1.5)
Loss of consciousness						
Coma (GCS: 3–8)	25,742	(2.4)	1,557	(3.8)	24,185	(2.3)
Not coma (GCS: 9–15)	10,53,108	(97.6)	39,583	(96.2)	10,13,525	(97.7)
Body temperature						
<34°C	2,157	(0.2)	88	(0.2)	2,069	(0.2)
34.0–34.9°C	2,748	(0.3)	87	(0.2)	2,661	(0.3)
35.0–35.9°C	1,11,346	(10.3)	3,365	(8.2)	1,07,981	(10.4)
36.0–36.9°C	6,53,873	(60.6)	22,007	(53.5)	6,31,866	(60.9)
37.0–37.9°C	1,95,335	(18.1)	9,254	(22.5)	1,86,081	(17.9)
38.0–38.9°C	63,574	(5.9)	3,778	(9.2)	59,796	(5.8)
≥39.0°C	49,817	(4.6)	2,561	(6.2)	47,256	(4.6)
Time of day						
Daytime (9:00–17:59)	5,24,581	(48.6)	13,853	(33.7)	5,10,728	(49.2)
Nighttime (0:00–8:59, 18:00–23:59)	5,54,269	(51.4)	27,287	(66.3)	5,26,982	(50.8)
Day of the week						
Weekday	7,69,644	(71.3)	27,578	(67.0)	7,42,066	(71.5)
Weekends	3,09,206	(28.7)	13,562	(33.0)	2,95,644	(28.5)
Location, *n* (%)						
Home	6,96,057	(64.5)	25,560	(62.1)	6,70,497	(64.6)
Public space	1,91,160	(17.7)	8,586	(20.9)	1,82,574	(17.6)
Workspace	26,907	(2.5)	587	(1.4)	26,320	(2.5)
Road	1,52,826	(14.2)	5,900	(14.3)	1,46,926	(14.2)
Other	11,900	(1.1)	507	(1.2)	11,393	(1.1)
Reason for ambulance call, *n* (%)						
Fire accident	843	(0.1)	70	(0.2)	773	(0.1)
Natural disaster	34	(0.0)	2	(0.0)	32	(0.0)
Water accident	57	(0.0)	1	(0.0)	56	(0.0)
Traffic accident involving car, ship, or aircraft	82,901	(7.7)	2,410	(5.9)	80,491	(7.8)
Injury, poisoning, and disease due to industrial accident	10,641	(1.0)	294	(0.7)	10,347	(1.0)
Disease and injury due to sports	5,342	(0.5)	140	(0.3)	5,202	(0.5)
Other injury	1,80,226	(16.7)	7,267	(17.7)	1,72,959	(16.7)
Trauma due to assault	6,058	(0.6)	690	(1.7)	5,368	(0.5)
Self-induced injury	6,059	(0.6)	1,110	(2.7)	4,949	(0.5)
Acute disease	7,86,416	(72.9)	29,146	(70.8)	7,57,270	(73.0)
Other	273	(0.0)	10	(0.0)	263	(0.0)
COVID-19 pandemic periods, *n* (%)						
Pre-pandemic period (2019/01/01–2020/01/28)	4,25,768	(39.5)	12,062	(29.3)	4,13,706	(39.9)
1st wave (2020/01/29–2020/06/13)	1,21,247	(11.2)	4,646	(11.3)	1,16,601	(11.2)
2st wave (2020/06/14–2020/10/09)	1,17,787	(10.9)	4,123	(10.0)	1,13,664	(11.0)
3st wave (2020/10/10–2021/02/28)	1,27,181	(11.8)	6,340	(15.4)	1,20,841	(11.6)
4st wave (2021/03/01–2021/06/20)	97,417	(9.0)	5,652	(13.7)	91,765	(8.8)
5st wave (2021/06/21–2021/12/16)	1,74,174	(16.1)	7,731	(18.8)	1,66,443	(16.0)
6st wave (2021/12/17–2021/12/31)	15,276	(1.4)	586	(1.4)	14,690	(1.4)

GCS, Glasgow Coma Scale; IQR, interquartile range.

Table 2 shows the factors associated with DIH during the COVID-19 pandemic. In this study, the factors associated with DIH were as follows: older adults (AOR: 1.226, [95% CI: 1.199–1.254]), coma (AOR: 1.333, [95% CI: 1.263–1.408]), nighttime (AOR: 1.975, [95% CI: 1.933–2.018]), weekends (AOR: 1.223, [95% CI: 1.198–1.250]), public space (AOR: 1.323, [95% CI: 1.289–1.358]), and road (AOR: 1.376, [95% CI: 1.326–1.429]). The SpO_2_ values associated with DIH were 91–95% (AOR: 1.147, [95% CI: 1.114–1.182]), 86–90% (AOR: 1.476, [95% CI: 1.400–1.555]), 81–85% (AOR: 1.584, [95% CI: 1.459–1.721]), and 80% or less (AOR: 1.636, [95% CI: 1.532–1.748]). For BT, they were 37.0–37.9°C (AOR: 1.506 [95% CI: 1.468–1.545]), 38.0–38.9°C (AOR: 1.969 [95% CI: 1.897–2.043]), and 39°C or higher (AOR: 1.912 [95% CI: 1.829–1.998]). The most relevant epidemic period of COVID-19 was the 4th wave (AOR: 2.134, [95% CI: 2.065–2.205]), followed by the 3rd wave (AOR: 1.842, [95% CI: 1.785–1.901]).

**Table 2:** Factors associated with difficulty in hospital acceptance during the COVID-19 pandemic in 2020–2021.

	Difficulty in hospital acceptance		Adjusted OR (95% CI)		*p* value	
	% (*n*/*N*)					
Age groups, *n* (%)						
Children (0–14 years old)	1.4	(956/68,765)	0.315	(0.295–0.337)	<	0.001
Adult (≥18 years, <65 years)	3.6	(22,825/625,431)	Reference			
Older adult (≥65 years)	4.5	(17,359/384,654)	1.226	(1.199–1.254)	<	0.001
Sex, *n* (%)						
Male	4.0	(21,879/543,760)	Reference			
Female	3.6	(19,261/535,090)	0.888	(0.870–0.906)	<	0.001
Saturation of oxygen (SpO_2_)						
96–100%	3.6	(31,740/889,012)	Reference			
91–95%	4.4	(6,003/135,056)	1.147	(1.114–1.182)	<	0.001
86–90%	6.0	(1,704/28,627)	1.476	(1.400–1.555)	<	0.001
81–85%	6.4	(643/10,021)	1.584	(1.459–1.721)	<	0.001
≤80%	6.5	(1,050/16,134)	1.636	(1.532–1.748)	<	0.001
Loss of consciousness						
Coma (GCS: 3–8)	6.0	(1,557/25,742)	1.333	(1.263–1.408)	<	0.001
Not coma (GCS: 9–15)	3.8	(39,583/1,053,108)	Reference			
Body temperature						
<34°C	4.1	(88/2,157)	0.989	(0.796–1.230)		0.924
34.0–34.9°C	3.2	(87/2,748)	0.889	(0.716–1.103)		0.284
35.0–35.9°C	3.0	(3,365/111,346)	0.894	(0.862–0.928)	<	0.001
36.0–36.9°C	3.4	(2,2007/653,873)	Reference			
37.0–37.9°C	4.7	(9,254/195,335)	1.506	(1.468–1.545)	<	0.001
38.0–38.9°C	5.9	(3,778/63,574)	1.969	(1.897–2.043)	<	0.001
≥39.0°C	5.1	(2,561/49,817)	1.912	(1.829–1.998)	<	0.001
Time of day						
Daytime (9:00–17:59)	2.6	(13,853/524,581)	Reference			
Nighttime (0:00–8:59, 18:00–23:59)	4.9	(27,287/554,269)	1.975	(1.933–2.018)	<	0.001
Day of the week						
Weekday	4.4	(13,562/309,206)	Reference			
Weekends	3.6	(27,578/769,644)	1.223	(1.198–1.250)	<	0.001
Location, *n* (%)						
Home	3.7	(25,560/696057)	Reference			
Public space	4.5	(8,586/191160)	1.323	(1.289–1.358)	<	0.001
Workspace	2.2	(587/26907)	0.694	(0.633–0.760)	<	0.001
Road	3.9	(5,900/152826)	1.376	(1.326–1.429)	<	0.001
Other	4.3	(507/11900)	1.407	(1.282–1.543)	<	0.001
Reason for ambulance call, *n* (%)						
Fire accident	8.3	(70/773)	2.521	(1.967–3.232)	<	0.001
Natural disaster	5.9	(2/32)	2.279	(0.543–9.558)		0.260
Water accident	1.8	(1/56)	0.409	(0.056–2.972)		0.377
Traffic accident involving car, ship, or aircraft	2.9	(2,410/82,901)	0.702	(0.665–0.741)	<	0.001
Injury, poisoning, and disease due to industrial accident	2.8	(294/10,641)	1.114	(0.980–1.267)		0.098
Disease and injury due to sports	2.6	(140/5,342)	0.859	(0.724–1.020)		0.083
Other injury	4.0	(7,267/180,226)	1.294	(1.258–1.331)	<	0.001
Trauma due to assault	11.4	(690/6,058)	2.666	(2.454–2.897)	<	0.001
Self-induced injury	18.3	(1,110/6.059)	5.566	(5.195–5.963)	<	0.001
Acute disease	3.7	(29,146/786,416)	Reference			
Other	3.7	(10/273)	1.006	(0.532–1.902)		0.985
COVID-19 pandemic periods, *n* (%)						
Pre-pandemic period (2019/01/01–2020/01/28)	2.8	(12,062/425,768)	Reference			
1st wave (2020/01/29–2020/06/13)	3.8	(4,646/121,247)	1.375	(1.328–1.423)	<	0.001
2st wave (2020/06/14–2020/10/09)	3.5	(4,123/117,787)	1.208	(1.165–1.252)	<	0.001
3st wave (2020/10/10–2021/02/28)	5.0	(6,340/127,181)	1.842	(1.785–1.901)	<	0.001
4st wave (2021/03/01–2021/06/20)	5.8	(5,652/97,417)	2.134	(2.065–2.205)	<	0.001
5st wave (2021/06/21–2021/12/16)	4.4	(7,731/174,174)	1.577	(1.532–1.624)	<	0.001
6st wave (2021/12/17–2021/12/31)	3.8	(586/15,276)	1.411	(1.296–1.536)	<	0.001

GCS, Glasgow Coma Scale; OR, odds ratio; CI, confidence interval.

Table 3 shows the factors associated with DIH in the pre-pandemic period, the 1st wave, when COVID-19 was first prevalent, and the 4th wave, when cases with DIH occurred most frequently. The AOR for SpO_2_ < 80% was 1.333 (95% CI: 1.157–1.536), whereas the AORs were 1.649 (95% CI: 1.365–1.991) in the 1st wave and 2.221 (AOR: 1.919–2.570) in the 4th wave. The AOR of 39°C or higher was 1.104 (95% CI: 1.002–1.216), whereas the AORs were 3.092 (95% CI: 2.732–3.499) in the 1st wave and 2.360 (AOR: 2.109–2.642) in the 4th wave.

**Table 3:** Factors associated with difficulty in hospital acceptance during the COVID-19 pandemic in 2020–2021 by pandemic period.

	Pre-pandemic (2019.1.1–2020.1.28)				1st wave (2020.1.29–2020.6.13)				4th wave (2021.3.1–2021.6.20)			
	Difficulty in hospital acceptance, % (*n*/*N*)		Adjusted OR (95% CI)		Difficulty in hospital acceptance, % (*n*/*N*)		Adjusted OR (95% CI)		Difficulty in hospital acceptance, % (*n*/*N*)		Adjusted OR (95% CI)	
Age groups, *n* (%)												
Children (0–14 years old)	1.2	(368/31,512)	0.409	(0.367–0.456)	1.4	(92/6,683)	0.285	(0.230–0.352)	1.6	(104/6,687)	0.204	(0.167–0.249)
Adult (≥18 years, <65 years)	2.6	(6,236/242,800)	Reference		3.6	(2,577/71,463)	Reference		5.9	(3,365/56,573)	Reference	
Older adult (≥65 years)	3.6	(5,458/151,456)	1.282	(1.231–1.335)	4.6	(1,977/43,101)	1.346	(1.259–1.439)	6.4	(2,183/34,157)	1.163	(1.094–1.236)
Sex, *n* (%)												
Male	3.0	(6,413/214,018)	Reference		4.0	(2,484/61,581)	Reference		5.9	(2,931/49,343)	Reference	
Female	2.7	(5,649/211,750)	0.880	(0.848–0.913)	3.6	(2,162/59,666)	0.902	(0.849–0.959)	5.7	(2,739/48,074)	0.965	(0.913–1.020)
Saturation of oxygen (SpO_2_)												
96–100%	2.8	(9,876/353,431)	Reference		3.5	(3,570/100,975)	Reference		5.1	(3,968/78,322)	Reference	
91–95%	2.9	(1,472/51,607)	1.071	(1.011–1.134)	4.7	(652/13,925)	1.151	(1.051–1.259)	7.6	(970/12,762)	1.293	(1.197–1.397)
86–90%	3.3	(366/10,986)	1.305	(1.170–1.457)	6.7	(216/3,231)	1.569	(1.351–1.822)	10.6	(343/3,236)	1.690	(1.495–1.911)
81–85%	3.6	(135/3,777)	1.377	(1.155–1.642)	6.8	(79/1,166)	1.574	(1.241–1.997)	10.9	(133/1,223)	1.760	(1.458–2.125)
≤80%	3.6	(213/5,967)	1.333	(1.157–1.536)	6.6	(129/1,950)	1.649	(1.365–1.991)	12.7	(238/1,874)	2.221	(1.919–2.570)
Loss of consciousness												
Coma (GCS: 3–8)	4.2	(424/10,118)	1.353	(1.220–1.499)	5.5	(169/3,085)	1.127	(0.956–1.329)	5.7	(5,439/95,028)	1.234	(1.062–1.434)
Not coma (GCS: 9–15)	2.8	(11,638/415,650)	Reference		3.8	(4,477/118,162)	Reference		8.9	(213/2,389)	Reference	
Body temperature												
<34°C	3.6	(25/704)	1.144	(0.762–1.717)	2.6	(6/227)	0.778	(0.343–1.767)	4.5	(11/220)	0.782	(0.421–1.453)
34.0–34.9°C	1.9	(20/1,077)	0.644	(0.412–1.005)	4.0	(15/371)	1.327	(0.788–2.235)	4.2	(10/223)	0.816	(0.429–1.551)
35.0–35.9°C	2.7	(1,292/47,007)	0.962	(0.905–1.021)	2.5	(348/13,925)	0.821	(0.732–0.921)	4.7	(388/9,286)	0.856	(0.768–0.955)
36.0–36.9°C	2.9	(7,365/256,953)	Reference		3.0	(2,285/75,493)	Reference		5.0	(2,767/58,562)	Reference	
37.0–37.9°C	2.9	(2,127/73,626)	1.101	(1.047–1.157)	5.7	(1,138/20,130)	2.015	(1.871–2.170)	7.7	(1,382/17,848)	1.711	(1.597–1.832)
38.0–38.9°C	3.0	(743/25,139)	1.252	(1.157–1.355)	7.9	(505/6,376)	2.947	(2.653–3.272)	10.1	(654/6,462)	2.196	(1.998–2.413)
≥39.0°C	2.3	(490/21,262)	1.104	(1.002–1.216)	7.4	(349/4,725)	3.092	(2.732–3.499)	9.1	(440/4,816)	2.360	(2.109–2.642)
Time of day												
Daytime (9:00–17:59)	1.7	(3,497/202,737)	Reference		2.7	(1,576/58,051)	Reference		4.3	(2,105/48,566)	Reference	
Nighttime (0:00–8:59, 18:00–23:59)	3.8	(8,565/223,031)	2.276	(2.184–2.371)	4.9	(3,070/63,196)	1.859	(1.743–1.981)	7.3	(3,547/48,851)	1.833	(1.730–1.942)
Day of the week												
Weekdays	2.6	(7,961/301,238)	Reference		3.6	(3,118/87,145)	Reference		5.5	(3,859/69,839)	Reference	
Weekends	3.3	(4,101/124,530)	1.230	(1.184–1.279)	4.5	(1,528/34,102)	1.236	(1.160–1.317)	6.5	(1,793/27,578)	1.182	(1.115–1.254)
Location, *n* (%)												
Home	2.6	(7,081/270,444)	Reference		3.7	(2,982/80,236)	Reference		5.7	(3,651/63,934)	Reference	
Public space	3.3	(2,627/79,270)	1.365	(1.302–1.431)	4.8	(964/19,883)	1.327	(1.227–1.434)	7.3	(1,225/16,815)	1.304	(1.215–1.400)
Workspace	1.5	(159/10,393)	0.661	(0.556–0.786)	2.1	(61/2,895)	0.609	(0.457–0.810)	3.0	(73/2,443)	0.666	(0.514–0.862)
Road	3.3	(2,032/60,930)	1.399	(1.312–1.493)	3.5	(583/16,894)	1.288	(1.147–1.446)	4.8	(633/13,073)	1.326	(1.185–1.482)
Other	3.4	(163/4,731)	1.383	(1.176–1.626)	4.2	(56/1,339)	1.563	(1.183–2.064)	6.1	(70/1,152)	1.554	(1.207–2.001)
Reason for ambulance call, *n* (%)												
Fire accident	7.7	(27/350)	3.045	(2.048–4.528)	5.2	(5/96)	1.546	(0.623–3.836)	10.4	(8/77)	2.040	(0.968–4.298)
Natural disaster	7.7	(1/13)	3.669	(0.471–28.591)	–	–	–		–	–	–	
Water accident	0	(0/19)	–		0	(0/7)	–		0	(0/4)	–	
Traffic accident involving car, ship, or aircraft	2.5	(827/32,476)	0.795	(0.726–0.872)	2.4	(222/9,078)	0.642	(0.561–0.763)	3.4	(255/7,404)	0.579	(0.491–0.682)
Injury, poisoning, and disease due to industrial accident	2.0	(85/4,223)	1.099	(0.869–1.389)	3.3	(38/1,137)	1.422	(0.989–2.045)	3.4	(30/892)	0.957	(0.643–1.426)
Disease and injury due to sports	2.0	(5/253)	0.889	(0.670–1.179)	3.4	(8/232)	1.062	(0.519–2.176)	4.5	(17/376)	1.022	(0.620–1.684)
Other injury	3.6	(2,533/69,811)	1.559	(1.485–1.637)	3.7	(774/20,882)	1.249	(1.146–1.361)	5.2	(822/15,868)	1.085	(1.000–1.179)
Trauma due to assault	10.4	(258/2,489)	3.044	(2.660–3.484)	11.9	(91/767)	2.773	(2.201–3.492)	10.6	(50/473)	1.598	(1.181–2.161)
Self-induced injury	14.8	(322/2,169)	5.763	(5.089–6.527)	16.5	(115/698)	5.001	(4.049–6.175)	22.8	(129/567)	4.891	(3.981–6.010)
Acute disease	2.6	(7,953/311,541)	Reference		3.8	(3,393/88,324)	Reference		6.0	(4,339/71,732)	Reference	
Other	3.4	(5/147)	1.253	(0.511–3.072)	0	(0/26)	–		8.3	(2/22)	1.394	(0.322–6.041)

GCS, Glasgow Coma Scale; OR, odds ratio; CI, confidence interval.

Figure 1 shows the mortality among the cases with DIH during each epidemic period. The highest mortality occurred in the 4th wave (3.9%, 219/5652), followed by the 3rd wave (3.5%, 224/6340).

## Discussion

In this study, we revealed that factors related to COVID-19, such as BT and SpO_2_, and the COVID-19 epidemic period were associated with DIH during the COVID-19 pandemic period in Osaka Prefecture, Japan. Furthermore, we found differences in the influence of each variable during the epidemic periods of COVID-19. This study, which analyzed a population-based emergency patient registry to assess the impact of emerging infectious disease on the EMS system, may help to examine the impact of the spread of new emerging infectious diseases on the healthcare system in future.

The epidemic period was associated with DIH, and the 4th wave was most associated with DIH in this study. Considering that the number of COVID-19 patients peaked in the 5th wave in Osaka Prefecture (11), it was likely that social confusion in the early phase of the pandemic and the large number of COVID-19 patients were not the only factors associated with the DIH. Park et al. reported a prolonged prehospital time for all patients, excluding those with out-of-hospital cardiac arrest, during the COVID-19 pandemic in 2020 (19). In a study of patients with ischemic stroke, Velasco et al. reported prolonged time from ambulance dispatch to hospital arrival during the pandemic period (20). Furthermore, a systemic review of prehospital care for patients with suspected stroke or transient ischemic attack (TIA) revealed that transport delay for patients with suspected stroke or TIA increased during the COVID-19 pandemic (21). In contrast, a systemic review of severe trauma during the restriction policy period in the first wave of the COVID-19 pandemic reported that the number of severe trauma patients decreased during this wave of the COVID-19 pandemic, but severity and mortality remained the same (22). Another study analyzing a multicenter trauma registry in Japan reported that time related to prehospital care increased during the pandemic in 2020, but in-hospital mortality did not change (23). As more patient occupy treatment space at medical facilities, emergency patients will have to be transported by ambulance to distant medical facilities because the facilities will not be able to accept excess patients. The COVID-19 pandemic may have limited the number of medical facilities accepting emergency patients because of the need for precautions against infection among healthcare workers. Based on these results and reports of previous studies, the number of patients with severe pneumonia and respiratory failure requiring ventilation and ECMO exploded in the 4th wave of this study, even among relatively young patients, due to a genetic alteration in the COVID-19 virus. As a result, emergency and critical care centers with intensive care units (ICUs) were permanently full and could not accept new emergency patients. In fact, according to the ECMO net open data source, the number of patients on ECMO in Osaka Prefecture increased in the 4th wave (24). After the 5th wave, when the infectivity of the COVID-19 virus increased but the rate of severe COVID-19 patients decreased, the rate of DIH decreased. It may thus be necessary to plan in advance or modify the medical system as appropriate to accommodate and discharge severe patients in order to smoothly accept emergency patients in the event of an unknown infectious disease pandemic in the future.

Secondarily, the analysis by epidemic period showed that the OR for fever was higher in the 1st wave than in the 4th wave. In the early stage of the COVID-19 pandemic, the route of infection and symptoms were unknown, and even nonspecific symptoms such as fever required suspicion of COVID-19 infection. In addition, because the route of infection and infectivity were unknown, healthcare workers were required to take very strict infection control measures. However, lockdowns were conducted in many countries around the world, and the distribution of infection prevention equipment was also halted. As a result, many medical facilities were unable to accommodate patients due to a shortage of infection prevention equipment and insufficient infection control measures. Indeed, there was a lack of infection prevention equipment during the COVID-19 pandemic in Japan (25). The risk of the spread of unknown infectious diseases will continue to exist, and it will be necessary for medical institutions and governments to stockpile sufficient infection prevention equipment against pandemic risk.

For SpO_2_, the OR was higher in the 4th wave than in the 1st wave. This may be due to the fact that the number of patients with severe respiratory failure requiring ventilators or ECMO increased as a result of the increased risk of severe illness caused by virus mutation, and ICUs for ventilating patients were permanently full. Indeed, in a registry study of several trauma patients in the Netherlands, the peak of COVID-19 patients had a negative impact on trauma care in that fewer severe trauma patients were admitted to the ICUs and worse outcomes were experienced, especially for patients with mild-to-moderate head trauma (26). Unlike with infection control equipment, ICUs cannot be stockpiled for future outbreaks. It would also not be economically feasible to maintain a constant reserve of ICUs in case of a pandemic. Therefore, during an infectious disease pandemic, it is necessary to convert ordinary ICUs into beds for severely infected patients. However, if the number of severely ill patients exceeds the capacity of ICUs, triage by doctors may be necessary to accommodate the patients in the ICUs or it may be necessary to have a system in which many physicians coordinate and decide where to transport patients. In the future, it will be necessary to establish a triage algorithm that included ethical considerations regarding the appropriateness of ICU admission. In addition, self-injured patients were associated with DIH in this study. This may be related to socioeconomic and cultural factors. To reveal the effect of socioeconomic and cultural factors, we plan further studies in the future.

There are several limitations in this study. First, the ORION system could collect data on emergency patients transported to emergency hospitals and emergency critical care centers within Osaka Prefecture but not on emergency patients transported to medical institutions other than these emergency medical institutions in Osaka Prefecture or to medical institutions outside Osaka Prefecture because the ORION system is operated by Osaka Prefecture and cannot be expanded to areas outside of Osaka Prefecture. In addition, no data were collected on the prognosis of patients who were not transported and who were transferred to other medical institutions. Furthermore, we could not collect detailed medical history data, such as medications and pregnancy. In this analysis, socioeconomic status, such as patient income and educational background, could not be evaluated because no data exist. Finally, this study was an observational study and unknown confounding factors could not be evaluated.

## Conclusion

In this study, we identified factors associated with the DIH during the COVID-19 pandemic. As various factors are involved in the spread of an unknown infectious disease, it is necessary not only to plan in advance but also to take appropriate measures according to the situation in order to smoothly accept emergency patients.

## Data availability statement

The data analyzed in this study is subject to the following licenses/restrictions: The data that support the findings of this study are available from Osaka Prefectural government, but the availability of these data is restricted. Data cannot be shared publicly because of the Protection Ordinance for Personal Information in Osaka Prefecture. Requests to access these datasets should be directed to YK, orion13@hp-emerg.med.osaka-u.ac.jp.

## Ethics statement

The studies involving humans were approved by the Ethics Committee of Osaka University Graduate School of Medicine. The studies were conducted in accordance with the local legislation and institutional requirements. The ethics committee/institutional review board waived the requirement of written informed consent for participation from the participants or the participants’ legal guardians/next of kin because the ORION data is anonymized without specific personal data, such as patient name, date of birth, and address, the requirement of obtaining patients’ informed consent was waived.

## Author contributions

YK: Conceptualization, Investigation, Methodology, Project administration, Writing – original draft. KT: Conceptualization, Formal analysis, Methodology, Resources, Software, Visualization, Writing – review & editing. SN: Conceptualization, Data curation, Resources, Writing – review & editing. JT: Conceptualization, Data curation, Resources, Writing – review & editing. TH: Conceptualization, Data curation, Resources, Writing – review & editing. HD: Conceptualization, Data curation, Resources, Writing – review & editing. TK: Conceptualization, Data curation, Methodology, Resources, Software, Validation, Writing – original draft, Writing – review & editing. JO: Data curation, Funding acquisition, Resources, Supervision, Writing – review & editing. TM: Conceptualization, Data curation, Resources, Writing – review & editing.
